# Multiscale higher-order TV operators for L1 regularization

**DOI:** 10.1186/s40679-018-0061-x

**Published:** 2018-10-23

**Authors:** Toby Sanders, Rodrigo B. Platte

**Affiliations:** School of Mathematical and Statistical Sciences, Arizona State University, P.O. Box 871804, Tempe, AZ 85287-1804 USA

**Keywords:** Image reconstruction, Sparsity, $\ell _1$ regularization, Electron tomography

## Abstract

In the realm of signal and image denoising and reconstruction, $\ell _1$ regularization techniques have generated a great deal of attention with a multitude of variants. In this work, we demonstrate that the $\ell _1$ formulation can sometimes result in undesirable artifacts that are inconsistent with desired sparsity promoting $\ell _0$ properties that the $\ell _1$ formulation is intended to approximate. With this as our motivation, we develop a multiscale higher-order total variation (MHOTV) approach, which we show is related to the use of multiscale Daubechies wavelets. The relationship of higher-order regularization methods with wavelets, which we believe has generally gone unrecognized, is shown to hold in several numerical results, although notable improvements are seen with our approach over both wavelets and classical HOTV. These results are presented for 1D signals and 2D images, and we include several examples that highlight the potential of our approach for improving two- and three-dimensional electron microscopy imaging. In the development approach, we construct the tools necessary for MHOTV computations to be performed efficiently, via operator decomposition and alternatively converting the problem into Fourier space.

## Introduction

Over the past couple of decades, $\ell _1$ regularization techniques such as total variation have become increasingly popular methods for image and signal denoising and reconstruction problems. Along with TV [1], a large variety of approaches for similar $\ell _1$ regularization approaches have been proposed for an array of problems. Signal and image recovery methods continue to attract a great deal of interest due to the wide variety of potential applications and ever increasing means of various sensing mechanisms to acquire data. To name a few, synthetic aperture radar (SAR) [2, 3], magnetic resonance imaging (MRI) [4–6], electron tomography [7, 8], and inpainting [9, 10] are all image recovery applications that have advanced in part due to $\ell _1$ regularization methods, and in each case the approach can be tailored to the challenges that the particular application poses. With many problems such as two- and three-dimensional electron microscopy imaging, the challenge is often to acquire as little data as necessary due to possible damage of the subject being imaged or because of time constraints, driving the need for inverse methods that can achieve the absolute best results from very limited and noisy data.

The mathematical description of the general problem we are interested in is to recover a signal or image $f\in \mathbb {R}^N$, from noisy measurements *b* of the form $b = Af+\epsilon$, where $A\in \mathbb {R}^{m \times N}$ is some sensing matrix that approximates the physical model of the particular problem. For example, in electron tomography *f* is a 3D nano-structure, *b* is a collection of projected microscopy images/data of *f*, and *A* is the projection operator that relates *f* and *b*. Given these ingredients, the $\ell _1$ regularized solution is given by

1 $$\begin{aligned} f_{\mathrm{rec}} = \arg \min _f \Big \{ \Vert Af - b \Vert _2^2 + \lambda \Vert T f \Vert _1 \Big \} , \end{aligned}$$

where *T* is some sparsifying linear transform and $\lambda$ is a parameter that balances the effects of the data and regularization terms. The appropriateness of this approach is that some prior knowledge of the signal informs one that *Tf* is sparse, or approximately sparse, and that the formulation with the $\ell _1$ norm encourages such sparsity [11–13]. In many applications, some knowledge of the appropriate transform is available, particularly with images and for other signals, this knowledge is in the form of some “smoothness.”

In the case of TV, the sparsifying transform is given by $T : \mathbb {R}^N \rightarrow \mathbb {R}^{N-1}$, where $(Tf)_i = f_{i+1} - f_i$. The general idea for this approach is that the signal *f* is assumed to be piecewise constant with a few discontinuities, in which case *Tf* is sparse. If this is not precisely true, this approach still effectively reduces unwanted oscillations at the cost of the well documented asing effect [14, 15]. However, for more general piecewise smooth functions higher-order TV (HOTV) regularization methods are effective [14, 16, 17], and they do not suffer from the staircasing effects. In this case, the transform maps *f* to approximations of discrete derivatives of *f*, e.g., higher-order finite differences of *f*.

Another popular choice for *T* is wavelet transform [4, 18, 19]. For instance, such a transform can be written as $T : \mathbb {R}^N \rightarrow \mathbb {R}^{N}$, where $(Tf)_j = \langle f , \psi _j \rangle$ and $\psi _j$ are orthonormal so that $f = \sum _j \langle f , \psi _j \rangle \psi _j$. The idea here is that for appropriately smooth signals, most of the signal’s energy is captured in the few low-frequency, larger-scaled elements of the basis. Thus, most of the coefficients can be neglected, and thus a sparse approximation of *f* exists with respect to the basis.

### Discussion and contribution

The crux of general $\ell _1$ regularization methods is that recovering a signal with the most sparse representation, that is recovering the solution with the smallest so called $\ell _0$ norm, is often equivalent to its convexly relaxed variant of recovering the signal with the smallest $\ell _1$ norm, which is a field of study called compressed sensing (CS) [11–13]. Although convex $\ell _1$ optimization algorithms are useful in promoting sparsity, some small nonzero coefficients may still persist, an obvious sign that the assumptions needed for the exactness guarantees given by CS theory sometimes do not hold in practice. This observation is largely the original motivation of our present work in developing a multiscale HOTV approach related to multiscale wavelet regularization.

Much work has been devoted to understanding and developing sparsity promoting regularization methods, which are related to our current work. Numerous variants of higher-order TV methods have been proposed [14, 17, 20]. For example, in [20], the authors propose an edge detection operator that annihilates polynomials, which leads them to operators close to finite difference matrices. In [14], a combination of a TV regularizer with a quadratic second-order regularizer is developed in the continuous domain to eliminate staircasing effects. Likewise, several authors have shown that using some combination of first- and second-order methods to be beneficial [16, 21–23]. Unfortunately, since there are multiple regularization terms, these methods typically introduce additional parameters that need to be tuned. In terms of theory, it has been well documented that under certain conditions, TV and HOTV are equivalent to reconstruction with splines [24, 25], i.e., the solution of such methods recovers a piecewise polynomial with a sparse set of jumps.

TV denoising in particular has several very interesting equivalences. It is well known that TV denoising and other more general first-order denoising methods are equivalent to smoothing with a certain nonlinear diffusion models [26], a typical result of writing the equivalent Euler–Lagrange equations. Perhaps discussed less frequently and most related to the observations in our current work, TV denoising is equivalent to soft threshold denoising with the highest-frequency basis elements of the Haar wavelets [27, 28], in particular with the so-called cycle spinning [29]. In general, however, the main difference between these methods is that with TV, the smoothness analysis is limited to the finest scales, whereas wavelet regularizations promote function smoothness at multiple scales. A main contribution of this article is to expand further on the relationship between wavelets in $\ell _1$ regularization and those $\ell _1$ methods related to HOTV. In regards to extension of wavelets, a number of multidimensional generalizations have been invented including curvelets and shearlets [18, 30, 31], which are primarily used for sparse function approximation and improve the approximation rates in two- and three dimensions compared with their one-dimensional counterparts. In terms of application, TV and HOTV methods have been shown to be effective for multidimensional electron microscopy data processing [7–9, 32], and these data sets serve as strong evidence for the potential of improved imaging in these domains with our method.

The method we develop here is an alternative for HOTV regularization which we refer to as multiscale HOTV (MHOTV). In contrast to previous work, our approach considers combining both a multiscale approach and higher-order TV methods for the class of image reconstruction problems. The motivation for such an approach is in observable sub par results due to the relaxation of the sparsity promotion through the $\ell _1$ norm, contrary to the aforementioned results with splines [24, 25]. In light of this, we determined this calls for analysis of the function behavior at multiple scales. As can be deduced, this multiscale strategy is similar to the treatment of wavelets, and we argue that our approach is indeed related to the use of Daubechies wavelets, with the main divergence coming in the orthogonality and/or frame conditions prescribed by the wavelets. Orthogonality may be unnecessary for general $\ell _1$ regularization techniques, although fundamental to thresholding denoising techniques, and the relaxation of this condition in our approach allows for better localization of the transform. In the development of MHOTV, we carefully address the computational concerns associated with our approach through the use of both the FFT and operator decompositions. We are able to show through several numerical examples that MHOTV provides a notable improvement to the current alternatives, and in particular our method is highlighted for potential improvements to two- and three-dimensional electron microscopy imaging.

The organization of the remainder of the article is as follows. In “HOTV and multiscale generalizations” section, we define the HOTV operators and the corresponding multiscale generalizations, and we precisely define the MHOTV $\ell _1$ regularization model. We also motivate the approach via a numerical example, make the connection with Daubechies wavelets, and show initial improvements with our method. In “Fast calculation of MHOTV operators” section, we address the computational concerns associated with calculating MHOTV coefficients, devising two distinct ways that they can be calculated in an efficient manner. In “Application to multidimensional electron microscopy and tomography” section, our method is highlighted for two- and three-dimensional electron microscopy imaging, both for denoising and tomographic imaging, indicating overall improvements for imaging in these domains with our approach. “Quantitative results” section provides robust quantitative results that further confirm the improvements seen with the previous examples. All of the results indicate that MHOTV is an improvement to the original HOTV and the related Daubechies wavelets. Some proofs and definitions are provided in Appendix.

## HOTV and multiscale generalizations

As an alternative to TV regularization, general order TV methods have been shown to be effective for $\ell _1$ regularization [8, 14, 16, 20]. The TV transform can be thought of as a finite difference approximation of the first derivative, thus annihilating a function in locations where the function is a constant, i.e., a polynomial of degree zero. Likewise, higher-order TV transforms can be considered higher-order finite difference approximations to higher-order derivatives, thus annihilating the corresponding degree polynomials. With this in mind, we have the following definition:

### **Definition 1**

(*Finite differences*) Let $\phi _k \in \mathbb {R}^N$ be defined by

2 $$\begin{aligned} (\phi _k)_m = {\left\{ \begin{array}{ll} (-1)^{k} &{} \text{ if } \, m=0\\ 0 &{} \text{ if } \, 1\le m<N-k \\ (-1)^{k+m+N} {k \atopwithdelims ()N-m} &{} \text{ if } \, N-k \le m < N \end{array}\right. }. \end{aligned}$$

Then for $f\in \mathbb {R}^N$, the periodic *k*th order finite difference of *f* is given by

$$\begin{aligned} f*\phi _k, \end{aligned}$$

where $*$ denotes the discrete convolution.

### *Remark 1*

The convolution in this definition (and in general) can be represented by multiplication with a circulant matrix $\Phi _k$, where each row of $\Phi _k$ holds a shifted version of $\phi _k$. An example of the matrix in the case $k=2$ is given by

3 $$\begin{aligned} \Phi _2 = \left( \begin{array}{c c c c c c} 1 &{} -2 &{} 1 &{} 0 &{} \dots &{} 0\\ 0 &{} 1 &{} -2 &{} 1 &{} \dots &{} 0 \\ 0 &{} 0 &{} 1 &{} -2 &{} \dots &{} 0 \\ \vdots &{} &{} \ddots &{} &{} \dots &{} \vdots \\ 1 &{} 0 &{} \dots &{} &{} 1 &{} -2\\ -2 &{} 1 &{} \dots &{} &{} 0 &{} 1 \end{array}\right) . \end{aligned}$$

Note that this definition uses a periodic extension of *f* and can be ignored by dropping the last *k* rows of $\Phi _k$.

With this definition, the HOTV model can be said to recover

4 $$\begin{aligned} f_{rec} = \arg \min _f \Big \{\Vert Af - b\Vert _2^2 + \lambda \Vert \Phi _k f\Vert _1 \Big \}. \end{aligned}$$

Unfortunately, for many real-world imaging problems, the equivalence between $\ell _1$ and $\ell _0$ may not hold in practice, yet the $\ell _1$ regularization still tends to encourage favorable solutions. In terms of the sparsity promoting transform, this means that the transform of the recovered function may not be truly sparse, but most of the values are instead relatively close to zero. For HOTV, this means that a local Taylor expansion of the recovered function will still contain some small nonzero higher-order coefficients, yet essentially unobservable at the very local scale. In other words, at some point *t*, there exists a polynomial expansion of minimal degree of *f* given by

5 $$\begin{aligned} f(x) {\approx } \sum _{m=0}^M \alpha _m(t) \frac{(x-t)^m}{m!} , \end{aligned}$$

which holds for all *x* within some small interval *I* around the point *t*. Ideally, a solution given by the order *k* HOTV model recovers a solution so that the coefficients $\alpha _m(t)$ vanish for $m\ge k$. The $\ell _1$ model allows for these coefficients to remain, although very small, and the function still *appears* to essentially be a polynomial of degree less than *k*. However, when this behavior persists over many points at a larger scale, the result can be a function which looks more like a trigonometric polynomial rather than an algebraic one.

**Fig. 1:**
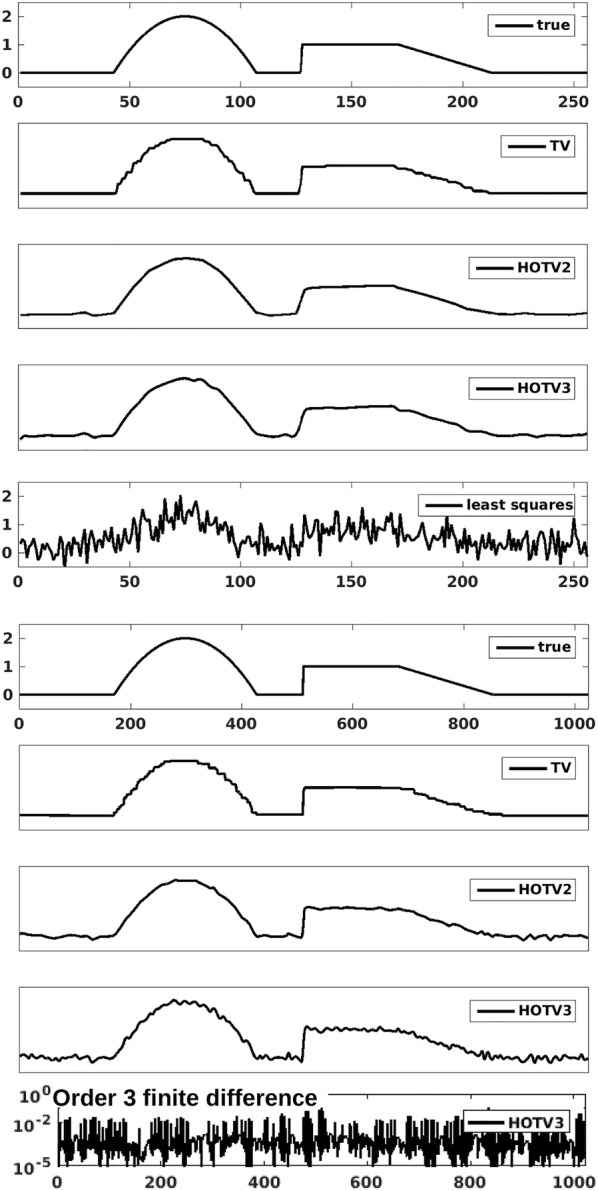
Rows 2–4 and 7–9 reconstruction of a piecewise polynomial function of degree two shown in the top row over 256 (top 5 plots) and 1024 (bottom 5 plots) points from random sampling at 50%. The corresponding least squares solution is shown in the fifth plot, and the third-order finite difference of the HOTV3 solution over the 1024 grid is shown on the bottom

This phenomenon is demonstrated in Fig. 1, where a piecewise polynomial of degree two was reconstructed from random noisy samples with 50% sampling^1^ using TV and HOTV regularizations. The sampling matrix $A\in \mathbb {R}^{N/2 \times N}$ is constructed so that a random 10% of its entries are set to be nonzero, where these nonzero values are uniformly distributed over [0, 1]. The samples were corrupted with normally distributed mean zero noise. Two different grid sizes are demonstrated, 256 and 1024, and it can be observed that these small oscillations become increasingly abundant with more grid points. However, in the bottom of the figure, the third-order finite difference of the HOTV3 solution plotted in logarithmic scale shows that locally this oscillatory behavior results in almost exact low-order polynomials, although *very* small amplitudes persist in the transformed domain, and thus not truly sparse in the $\ell _0$ sense. Nevertheless, all regularization approaches should still be deemed useful, as evidenced by the least squares solution shown.

Due to these phenomena, we propose a multiscale HOTV approach, which considers the regularization transform at multiple scales. The idea is that a larger stencil would penalize these oscillations even with the $\ell _1$ norm. As TV generalizes to the Haar wavelet by stretching and scaling of the elements, we propose the same with HOTV. To this end, we give the following definition.

### **Definition 2**

(*Multiscale finite differences*) Let $\phi _{k,j} \in \mathbb {R}^N$ be defined by

6 $$\begin{aligned}&(\phi _{k,j})_m \nonumber \\&\quad ={\left\{ \begin{array}{ll} (-1)^{k} &{} \text{ if } \, m=0\\ 0 &{} \text{ if } \, 1 \le m \le N-j(k+1)\\ (-1)^{k+\lfloor \frac{N-m}{j} \rfloor } {k \atopwithdelims ()\lfloor \frac{N-m}{j} \rfloor } &{} \text{ if } \, N-j(k+1)< m < N \end{array}\right. }. \end{aligned}$$

Then for $f\in \mathbb {R}^N$, the periodic *k*th order finite difference of scale *j* of *f* is given by

$$\begin{aligned} f*\phi _{k,j}, \end{aligned}$$

where $*$ denotes the discrete convolution.

### *Remark 2*

Again, this convolution can be represented as multiplication with a circulant matrix $\Phi _{k,j}$. An example of $\Phi _{k,j}$ in the case $k=2$ and $j=2$ is given in (7).

7 $$\begin{aligned} \Phi _{2,2} = \left( \begin{array}{c c c c c c c c c} 1 &{} 1 &{} -2 &{} -2 &{} 1 &{} 1 &{} 0 &{} \dots &{} 0\\ 0 &{} 1 &{} 1 &{} -2 &{} -2 &{} 1 &{} 1 &{} \dots &{} 0 \\ 0 &{} 0 &{} 1 &{} 1 &{} -2 &{} -2 &{} 1 &{} \dots &{} 0 \\ \vdots &{} &{} &{} \ddots &{} &{}&{} &{}\dots &{} \vdots \\ -2 &{} -2 &{} 1 &{} 1 &{} 0&{} 0 &{} \dots &{} 1 &{} 1\\ 1 &{} -2 &{}-2 &{} 1 &{} 1 &{} 0 &{} \dots &{} 0 &{} 1 \end{array}\right) . \end{aligned}$$

### MHOTV reconstruction model

We now present the general model for MHOTV reconstruction. Generally speaking, we still use the model presented in (1), where *A* maps the unknown function *f* to some perhaps noisy measurements given by *b*, from which we use to reconstruct *f*. Our sparsity promoting transforms are now given by the matrices $\Phi _{k,2^j}$, for $j=0,1,\dots , \ell$, where $\ell$ is the maximum scaling of the operator used and *k* is the chosen order. Setting our maximum scaling to $\ell =0$ corresponds to the traditional HOTV approach. Although not completely necessary, we choose a dyadic scaling of the operators, similar to the treatment of wavelets. As with wavelets, we will show that this is convenient for computational purposes. Finally then our reconstruction model is given by

8 $$\begin{aligned} f_{rec} = \arg \min _f \Big \{ \Vert A f - b\Vert _2^2 + \frac{\lambda }{\ell +1} \sum _{j=0}^\ell 2^{-(j+k-1)} \Vert \Phi _{k,2^j} f \Vert _1\Big \}. \end{aligned}$$

The factor of $2^{-j}$ is a normalization that accounts for the increasing norms of each operator, which would otherwise weigh too heavily to the largest scaling operator.^2^ The scaling of the parameter $\lambda$ by $\ell +1$ simplifies the selection of the parameter, which would otherwise need to be manually scaled by such a factor to account for the number of scales being used. By similar reasoning, the additional scaling by $2^{-k+1}$ is used to account for the order *k* of the method [33].

### Relationship to Daubechies wavelets

Wavelets can be characterized as an orthonormal basis that is generated through a multiresolutional analysis [19, 34]. The multiresolutional analysis leads to the shifting and dyadic stretching and scaling of a single generating mother wavelet, analogous to our treatment of MHOTV by shifting and stretching of a single row or element of the matrices $\Phi _k$. From this very general characterization, there are a number of parameters in the design of the wavelets. For Daubechies wavelets the smoothness is characterized by the number of vanishing moments, i.e., the number of polynomial orders to which the wavelet is orthogonal. A wavelet with *k* vanishing moments acts as a multiscale differential operator of order *k*. As a trade off, an increasing number of vanishing moments chosen for the wavelet basis results in an increase in the support of the wavelet functions, and Daubechies wavelets are designed to yield the orthonormal wavelet basis of minimal support given a selected number of vanishing moments [19].

To develop a basic mathematical description of a wavelet expansion, suppose we want to represent a *uniform pixelated* function with $2^n$ pixels on [0, 1] in terms of the wavelet basis. Then denoting our scaling function and mother wavelet with *k* vanishing moments by $\varphi _k$ and $\psi _k$, respectively, we have the following orthonormal wavelet representation

9 $$\begin{aligned} f = \sum _{t=0}^{2^{\ell } - 1} \langle f , \varphi _{k,\ell ,t} \rangle \varphi _{k,\ell ,t} + \sum _{j=\ell }^{n-1} \sum _{t=0}^{2^{j}-1} \langle f , \psi _{k,j,t} \rangle \psi _{k,j,t} . \end{aligned}$$

Here, $\psi _{k,j,t}(x) = 2^{j/2} \psi _k \left( 2^j x - t \right)$ and similarly for $\varphi _{k,j,t}$, i.e., shrinking and scaling of the generating wavelet functions. The parameter $\ell$ is a positive integer with $0\le \ell \le n$, and the value $n-\ell$ is said to be the number of *scales* in the wavelet expansion.^3^ With the representation in (9), the coefficients for the scaling functions in the first sum capture most of the energy of the signal, and the wavelet coefficients $c_{k,j,t} = \langle f,\psi _{k,j,t} \rangle$ *vanish* for values of *t* where *f* is a polynomial of degree $k-1$ over the support of $\psi _{k,j,t}$. For $\ell _1$ regularization, we only need to be concerned with regularization of the wavelet coefficients in (9), and thus the coefficients for the scaling functions in the first sum are not included in the regularization.

Connecting these ideas to HOTV, we see that these transforms are playing similar roles. Both are prescribed by the number of vanishing moments, or in the language of HOTV, the highest-order polynomial that is annihilated by the approach. Furthermore, both are designed to yield minimal support given the number of vanishing moments. The crucial difference lies in the orthogonality condition prescribed by wavelets, which further increases the support of the wavelet elements. We emphasize again that this condition is fundamental to compression and threshold denoising methods, but not necessarily useful with general image reconstruction problems.

Finally, one additional technique utilized for $\ell _1$ regularization and denoising as well is the use of a wavelet frame by taking all possible shifts for each scaling of the wavelets, which is sometimes referred to as translational invariant cycle spinning [29, 35, 36]. This eliminates the lack of translation invariance of a wavelet basis that can otherwise result in unwanted artifacts near discontinuities. With this in mind, we may define the wavelet frame elements by

$$\begin{aligned} {\tilde{\psi }}_{k,j,t}(x) = 2^{j/2} \psi _k \left( 2^j (x - t2^{-n})\right) , \quad t = 0 , 1 , \dots , 2^{n-1}. \end{aligned}$$

Then the *averaged* wavelet frame representation of *f* may be written as

$$\begin{aligned} f&= \sum _{t=0}^{2^{\ell } - 1} \langle f , \varphi _{k,\ell ,t} \rangle \varphi _{k,\ell ,t} + \sum _{j=\ell }^{n-1}2^{j-n} \sum _{t=0}^{2^{n}-1} \langle f , {\tilde{\psi }}_{k,j,t} \rangle {\tilde{\psi }}_{k,j,t} \\&= \sum _{t=0}^{2^{\ell } - 1} \langle f , \varphi _{k,\ell ,t} \rangle \varphi _{k,\ell ,t} + \sum _{j= \ell }^{n-1} 2^{j-n} \Psi _{k,j}^T (f * \psi _{k,j,0}(-x)), \end{aligned}$$

where $\Psi _{k,j}^T = ({\tilde{\psi }}_{k,j,0}, {\tilde{\psi }}_{k,j,1}, \dots , {\tilde{\psi }}_{k,j,2^n - 1})$. Hence, a wavelet approach promotes sparsity with respect to the vectors $f*\psi _{k,j,0}$, or equivalently with respect to $\Psi _{k,j}f$. Then, a regularization norm in this setting takes the form

10 $$\begin{aligned} \sum _{j=\ell }^{n-1} \Vert \Psi _{k,j} f \Vert _1, \end{aligned}$$

which is analogous to our regularization norm in (8). For wavelets, the scalings are inherent to function definitions, and the dyadic stretching of the elements is indicated by *j* as opposed to $2^j$.

The case when $\ell =n-1$ would be most closely related to the original HOTV, and for smaller values of $\ell$, the wavelets are more comparable to the MHOTV development in this article.

**Fig. 2:**
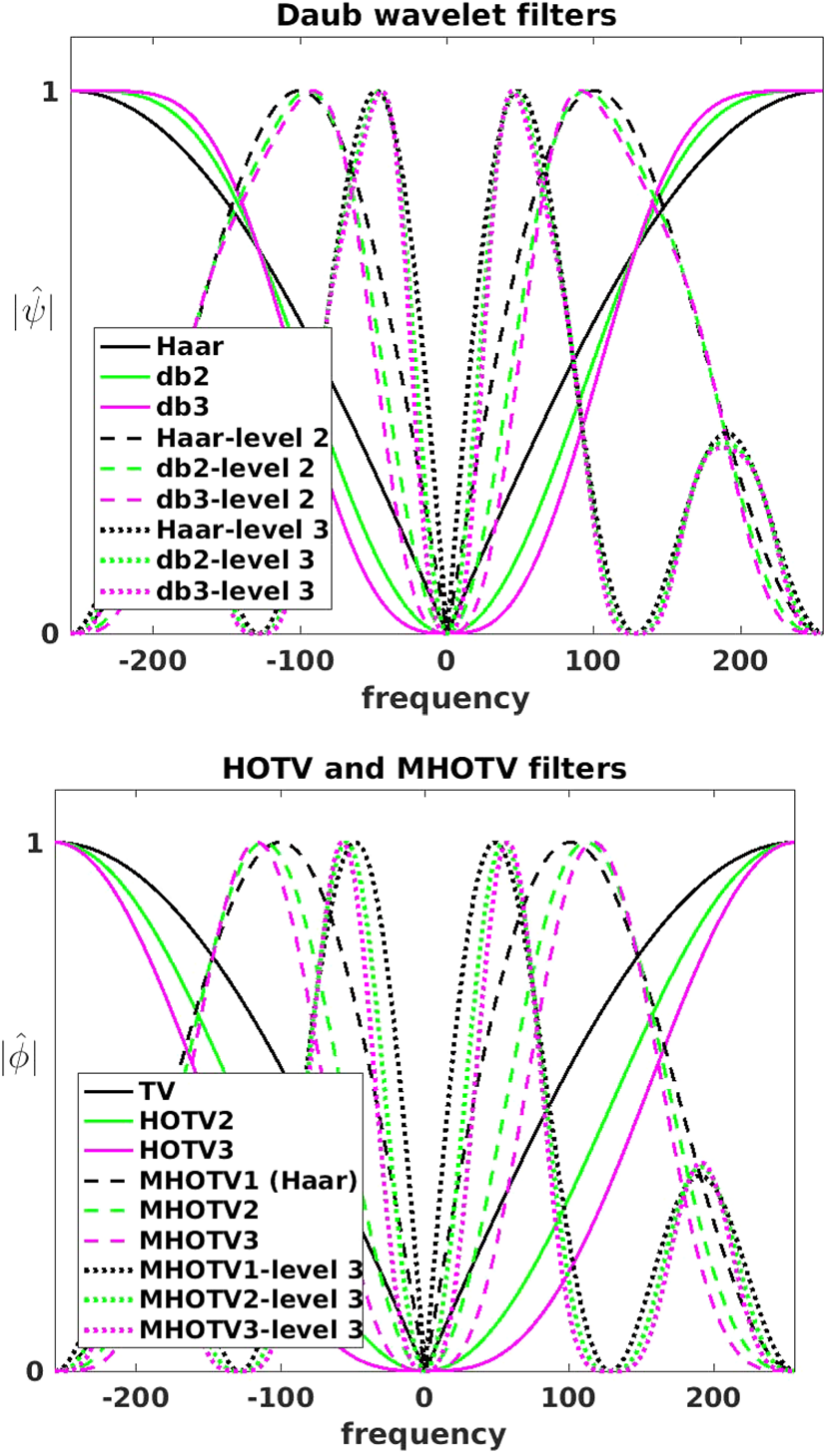
The filters in Fourier space of wavelet and MHOTV convolution functions

Since computing both MHOTV operators and wavelets coefficients are convolutional operations, we may visualize their corresponding filters in Fourier space, providing us another basis for comparison, which we have done in Fig. 2. Each of these curves can be interpreted as high-pass filters, where the higher levels pass increasingly lower frequencies. A very close similarity of the wavelet filters and MHOTV filters can be observed in Fig. 2, providing a strong visual confirmation to our preceding discussion on the close relationship between the two.

### Repeat of 1D simulations

**Fig. 3:**
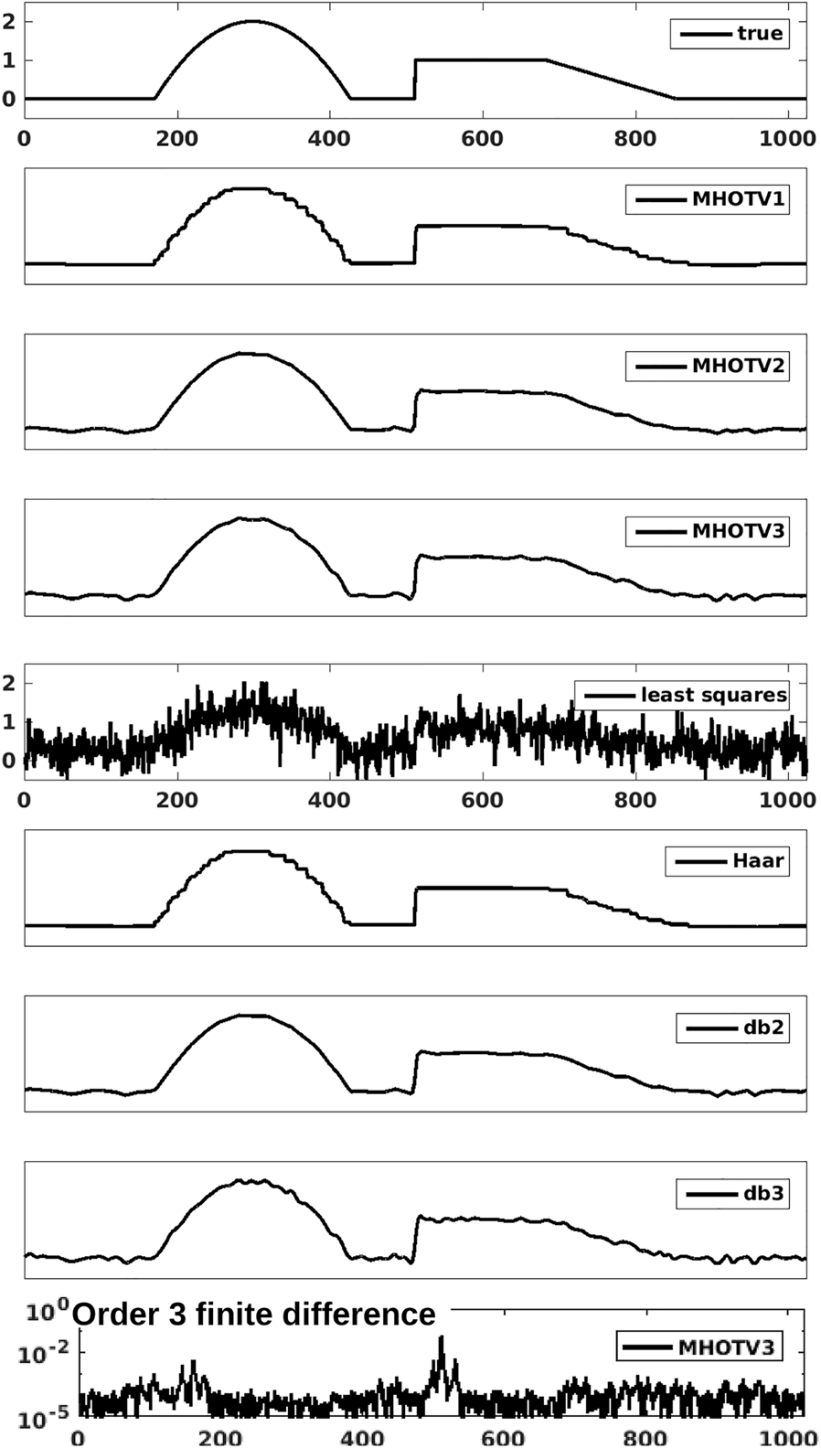
Reconstruction of a piecewise polynomial function of degree two over 1024 stencil from random sampling at 50%. Three scales are used for both the Daubechies wavelets and multiscale HOTV

To compare MHOTV and wavelet regularized reconstructions, we repeat the numerical examples presented in Fig. 1 with the same noisy data used for the HOTV reconstruction. The corresponding reconstruction with MHOTV and wavelets are presented in Fig. 3. Recall that the measurements were collected at a 50% sampling rate and corrupted with normally distributed mean zero noise. For the multiscale HOTV and wavelets, three scaling levels were used. The selection of the regularization parameter $\lambda$ was set to the same value for each order for HOTV and the wavelets, where we used a similar normalization approach for the wavelets coefficients as presented in (8).

The results in Fig. 3 were generated with orders 1, 2, and 3. The order is indicated with the numbers next to the approach in the legends, e.g., we denote the order $k=3$ MHOTV approach with MHOTV3. For a baseline comparison, the least squares solution is shown as well. Compared with the corresponding 1024 reconstructions from HOTV in Fig. 1, these solutions show clear improvements, particularly with the higher orders. As we expect, although the MHOTV1 and Haar wavelet coefficients are computed in a different manner, the resulting reconstruction is identical since the models are theoretically equivalent. They both exhibit the staircasing and noise effects in precisely the same locations. The higher-order approaches also show many similar effects of the noisy features, exhibiting certain oscillatory features with the same general behavior in precisely the same locations. However, with the higher orders, these approaches are not equivalent and MHOTV provides regulatory information at finer scales due to the minimal support of the transform elements. The result appears to be a modest improvement in the resulting reconstructions.

Finally, in the bottom of the figure, the third-order finite difference of the MHOTV3 solution is plotted in logarithmic scale. Comparing this with the original HOTV3 finite difference in Fig. 1, we observe that the solution exhibits much better sparsity with respect to this transform domain, as desired.

## Fast calculation of MHOTV operators

Calculation of traditional HOTV coefficients is a computationally inexpensive task, due to the sparsity of the matrix operator. However, with increasing dyadic scales the direct calculation increases exponentially. Due to this, in the proceeding section, we develop two distinct approaches which show that these calculations can be carried out with linear increase in the flop count with respect to the number of scales used.

Fast computation of standard HOTV can be done in several ways. One can construct the sparse matrix $\Phi _k$ and perform matrix computations directly, a calculation with runtime of *kN* flops. One could make use of other procedures, such as MATLAB’s “diff” command which requires the same flop count without storing the matrix. With MHOTV, these approaches become less appealing. With matrix construction, if one is using several scales, then several matrices need to be computed and stored, and the matrices become significantly less sparse for larger scales. The “diff” command cannot be implemented directly for larger-scale HOTV operators.

Another alternative is to use the Fourier convolution theorem to perform the convolution operation via a product in Fourier space. For the traditional HOTV operators, this can be fairly slow compared with the matrix and “diff” approach, since the necessary two discrete Fourier transforms would require $\sim 2 N\log _2 N$ flops compared with the *kN* flops for the alternative implementations. However, this method is relatively comparable for MHOTV, since the Fourier transforms only need to be computed once to determine the coefficients at all scales.

We outline two procedures for efficient calculation of MHOTV. First, we describe the Fourier approach, where we derive precise formulas for the MHOTV Fourier filters. Second, we describe an alternative efficient approach by decomposition of the MHOTV matrix operators.

### Computation via Fourier transforms

By the Fourier convolution theorem, the MHOTV operators can be computed as multiplications in Fourier space, i.e.,

11 $$\begin{aligned} f*\phi _{k,j} = F^{-1} \left( F(f)\cdot F(\phi _{k,j})\right) , \end{aligned}$$

where *F* denotes the discrete Fourier transform. Although this can be numerically computed, it is a convenient to have an exact formula for the discrete Fourier transform of $\phi _{k,j}$. Moreover, analytic determination of $F(\phi _{k,j})$ allows us to generalize the MHOTV to fractional orders.

#### **Proposition 1**

*The DFT of the vector* $\phi _{k,j}$* defined in* (6)* has an explicit expression given by*

12 $$\begin{aligned} F( \phi _{k,j} )_\xi = \frac{\left( e^{ \frac{i2\pi \xi j}{N}} - 1\right) ^{k+1}}{e^{\frac{i2\pi \xi }{N}}-1}, \end{aligned}$$

*for* $$\xi = 0, 1, \dots , N-1$$.

#### *Proof*

The expression for the $\xi$th Fourier coefficient in the DFT of $\phi _{k,j}$ is given by

13 $$\begin{aligned} F( \phi _{k,j} )_\xi = \sum _{m=0}^{N-1} (\phi _{k,j})_m e^{\frac{-i2\pi \xi }{N}m}. \end{aligned}$$

Notice that the terms $1\le m \le N - j(k+1)$ vanish by definition of $\phi _{k,j}$. For the latter terms, we make the substitution $n=N-m$ and flip the sum to give the expression

14 $$\begin{aligned} F(\phi _{k,j})_\xi = \sum _{n=0}^{j(k+1)-1} (-1)^{k + \lfloor {\frac{n}{j}}\rfloor } {k \atopwithdelims ()\lfloor \frac{n}{j} \rfloor } e^{\frac{-i2\pi \xi }{N}(N-n)}, \end{aligned}$$

where the term $n=0$ corresponds to $j=0$ and the following indices $n=1,2,\dots , m(k+1)-1$, correspond to $j=N-1,N-2, \dots , N-m(k+1)+1$, respectively. Notice that we may drop the *N* in the numerator of the exponential and that the values of $\phi _{k,j}$ repeat over strings of length *j*. Therefore, each of these corresponding strings of exponential terms in (13) get the same weights, leading to the following sum:

15 $$\begin{aligned} F( \phi _{k,j} )_\xi = \sum _{m=0}^k \left( (-1)^{m+k}{k\atopwithdelims ()m} \left[ \sum _{\ell =0}^{j-1} e^{\frac{i2\pi \xi }{N}(jm+\ell )} \right] \right) . \end{aligned}$$

Here, the inner sum represents the *j* consecutive terms in (13) that receive the same weights from $\phi _{k,j}$, namely $(-1)^{m+k}{k\atopwithdelims ()m}$. Switching the order of summation, we recognize the sum over *m* as a binomial expansion leading to

$$\begin{aligned} F( \phi _{k,j} )_\xi&= \sum _{\ell =0}^{j-1} \sum _{m=0}^k (-1)^{m+k}{k\atopwithdelims ()m} e^{\frac{i2\pi \xi }{N}(jm+\ell )}\\&= \sum _{\ell =0}^{j-1} \left( e^{\frac{i2\pi \xi }{N}j} - 1 \right) ^k e^{\frac{i2\pi \xi }{N}\ell }. \end{aligned}$$

The remainder of the proof follows by elementary calculations. $\square$

### Fast computation via operator decomposition

In this section, we give a decomposition for the matrix operator $\Phi _{k,2^j}$ and describe how this decomposition can be used for rapid calculation of MHOTV operators. The decomposition of $\Phi _{k,2^j}$ is given in the following theorem.

#### **Theorem 1**

*Let the matrix* $P_j$ *with entries* $\{p_{m,n}\}_{m,n=1}^N$ *be defined by*

16 $$\begin{aligned} p_{m,n} = {\left\{ \begin{array}{ll} 1 &{} \quad \text{ if } m=n\\ 1 &{} \quad \text{ if } n= (m+j-1)\bmod {(N)} + 1 \\ 0 &{} \quad \text{ if } {\mathrm{otherwise}} \end{array}\right. }. \end{aligned}$$

*Then the following holds:*

1. *The entries of* $P_j^{k+1}$,* which we denote by* $\{p_{m,n}(j,k)\}_{m,n=1}^N$,* are given by*
  $$\begin{aligned} p_{m,n}(j,k) = {\left\{ \begin{array}{ll} {k+1 \atopwithdelims ()\ell } &{}\text{ if } n = (m+j\ell -1)\bmod ({N)}+1\\ 0 &{}\text{ if } {\mathrm{otherwise}} \end{array}\right. }, \end{aligned}$$
  *where it is implied* $\ell$* is an integer satisfying* $0\le \ell \le k+1$.
2. $\Phi _{k,2^j}$ *has the decomposition*
  17 $$\begin{aligned} \Phi _{k,2^j} = P_j^{k+1} P_{j-1}^{k+1} \cdots P_1^{k+1} \Phi _k \end{aligned}$$
  * and therefore*
  18 $$\begin{aligned} \Phi _{k,2^j} = P_j^{k+1} \Phi _{k,2^{j-1}}. \end{aligned}$$
3. *The equality in* (17)* holds for any rearrangement of the product of matrices.*

The proof of this theorem is given in Appendix. The matrices $P_2$ and $P_2^2$ are shown below to illustrate the sparse structure of these operators:

$$\begin{aligned} P_2&= \left( \begin{array}{cccccc} 1 &{} 0 &{} 1 &{} 0 &{} \dots &{} 0\\ 0 &{} 1 &{} 0 &{} 1 &{} \dots &{} 0\\ \vdots &{} &{} \ddots &{} &{} \dots &{} \vdots \\ 0 &{} 1 &{} 0 &{} \dots &{} &{} 1 \end{array}\right) , \\ P_2^2&= \left( \begin{array}{ccccccc} 1 &{} 0 &{} 2 &{} 0 &{} 1 &{} \dots &{} 0\\ 0 &{} 1 &{} 0 &{} 2 &{} 0 &{} \dots &{} 0\\ \vdots &{} &{} \ddots &{} &{} &{} \dots &{} \vdots \\ 0 &{} 2 &{} 0 &{} 1 &{} 0 &{} \dots &{} 1 \end{array}\right) . \end{aligned}$$

#### **Proposition 2**

*Direct calculation of* $\Phi _{k,2^j}$* requires* $2^j Nk$* flops. The same calculation using the decomposition in* (17)* requires* $j N(k+1) + Nk$* flops. The same calculation using the Fourier method requires* $2N\log _2 N + N$.

Proposition 2 is a direct result of Theorem 1, the Fourier convolution theorem combined with the FFT, and the flops required for the direct calculation. We assume that the FFT and inverse FFT can be computed in $N\log _2 N$ flops, although the exact count is somewhat vague, depending on the precise algorithm and if *N* is a power of 2. To compute the full set of operators, we can get away with less flops and then adding the flops for each level. If we use the decomposition approach to calculate the operators as determined by (17), the associated computations are limited to that at the highest scale, since the intermediate scales are determined in this calculation as pointed out in (18). If we use the Fourier approach for calculating the coefficients in (8), only one forward FFT is required for the function *f*. Then, the product of *F*(*f*) and $F(\phi _{k,2^j})$ must be computed for each *j*, as well as the inverse FFT for each of these products. The observations lead to the following corollary.

#### **Corollary 1**

*Let T be the matrix containing the complete set of* $\ell +1$* operators involved in the MHOTV* $\ell _1$* regularization norm, so that* $T^T= [\Phi _{k,1}^T , \Phi _{k,2}^T , \dots , \Phi _{k,2^\ell }^T].$ *Then, calculating T using the operator decomposition given in Theorem* 1* requires* $\ell N(k+1) + Nk$ *flops. Calculating T using the Fourier approach requires a total flop count of* $(\ell +2) N\log _2 N+(\ell +1) N$.

A few concluding remarks are in order.

#### *Remark 3*

All of the results presented are for 1D signals. For higher dimensions say 2D, the operators can be applied along each row and column, and the flop count is only doubled, disregarding the likely increased number of indices.

#### *Remark 4*

To solve (8), we use the well-established alternating direction method of multipliers (ADMM) [37–39]. This approach introduces splitting variables that allows one to split the objective functional into equivalent subproblems that can be solved relatively fast. Our algorithm can be downloaded at [40], and some of the simulations in the proceeding section can also be found there.

## Application to multidimensional electron microscopy and tomography

In this section, we apply our proposed approach to nanoscale multidimensional electron microscopy data, for both denoising and tomographic reconstruction. The data used for our experiments are performed on one of the openly available tomographic tilt series data [41]. We use the data set labeled as “Tom_2”, which contains platinum nanoparticles embedded on a graphitized carbon nanofibre support. We apply our methods to these data in 2 ways: first as a 2D image denoising problem of a single projection and second as a 2D tomographic image reconstruction problem from the tilt series. These data serve as an excellent test case, since there are many projections with very high SNR, and we may test the accuracy of various methods by observing the results when we limit the quality of these data.

**Fig. 4:**
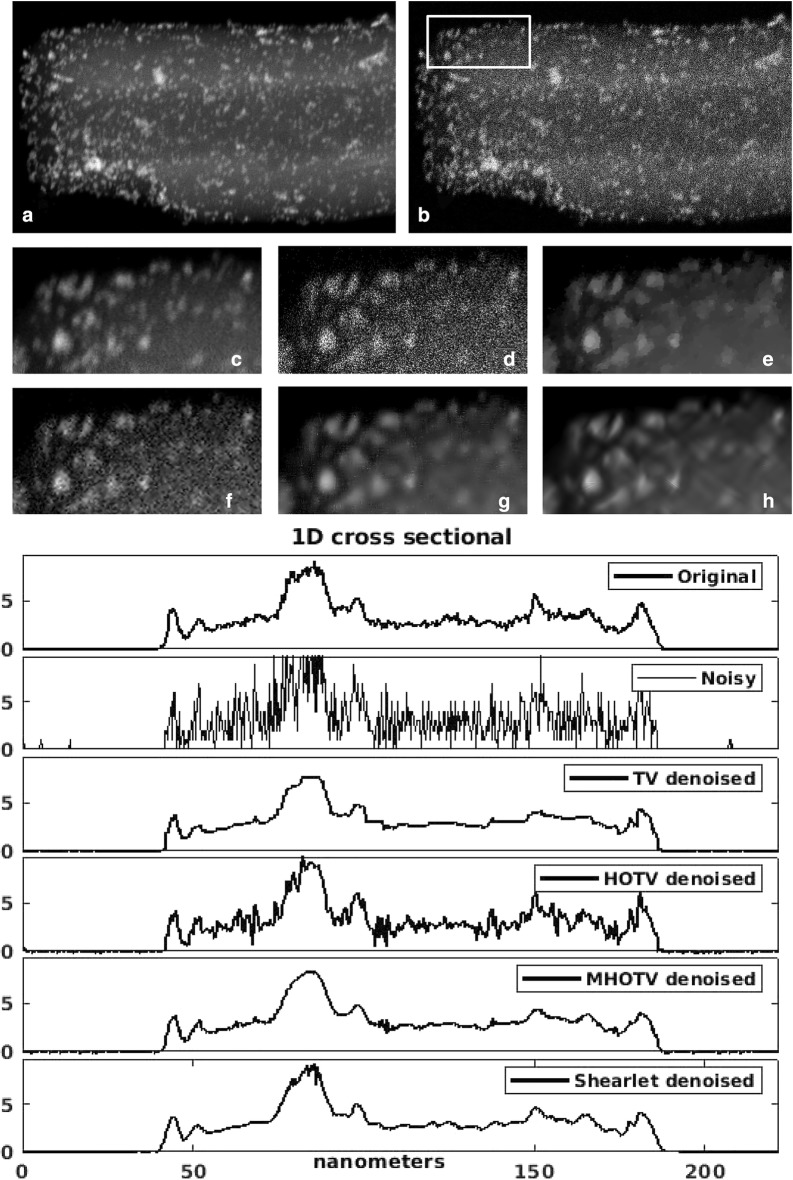
Denoising of an electron microscopy projection with Poisson noise. Small magnified patch is shown below for detailed analysis, where this patch is indicated in **b**. In **a** and **c** are the original image, with little noise, and in **b** and **d** are the simulated noisy images. In **e** is the TV denoised image, in **f** is the HOTV denoised, in **g** is the MHOTV denoised, and in **h** is the shearlet denoised by hard thresholding. One-dimensional plots of a single cross-section are shown on the right for an additional point of comparison

For the image denoising problem, we select one of the projection images from the full tilt series. These projection images have an excellent SNR, so we consider the original image as close to the ground truth. Therefore, a noisy version of the image was generated, where the usual Poisson noise model for noise in microscopy images is assumed. The image was scaled so that the mean values for the number of electrons per pixel, and hence the mean value for the Poisson noise, is 10, resulting in a maximum SNR of $\sqrt{10}$. The original and noisy images are shown in Fig. 4a, b, respectively. For closer inspection, a zoomed region (indicated in (b)) is shown in (c) and (d), and a one-dimensional cross section of the 2D images are plotted in the right panel of Fig. 4. To account for the Poisson noise, we use a weighted $\ell _2$ norm for the fidelity term in (1), similar to that used in [42].

The resulting denoised version showing only the small region are given in Fig. 4e–h, and the 1D plots are also shown in the right panel. In (e) is TV denoising, in (f) is HOTV denoising of order 3, and in (g) is our MHOTV denoising of order 3 with 3 levels. In (h) is the result of hard-thresholding with a shearlet frame [30, 31]. TV denoising is clearly not optimal for this type of image where an over-smoothing or over regularization occurs, since projection images most certainly do not abide by the general piecewise constant assumption, making higher-order methods more preferable [9]. On the other hand, due to the low SNR in the noisy image, with the HOTV denoised image, we again observe many unwanted oscillations. The MHOTV denoised image performs well in this regard, as well as the shearlet thresholding approach. Both retain much more of the image integrity than TV, while also eliminating most of the noise which is still present with HOTV. The one-dimensional plots in the right panel agree with these observations.

**Fig. 5:**
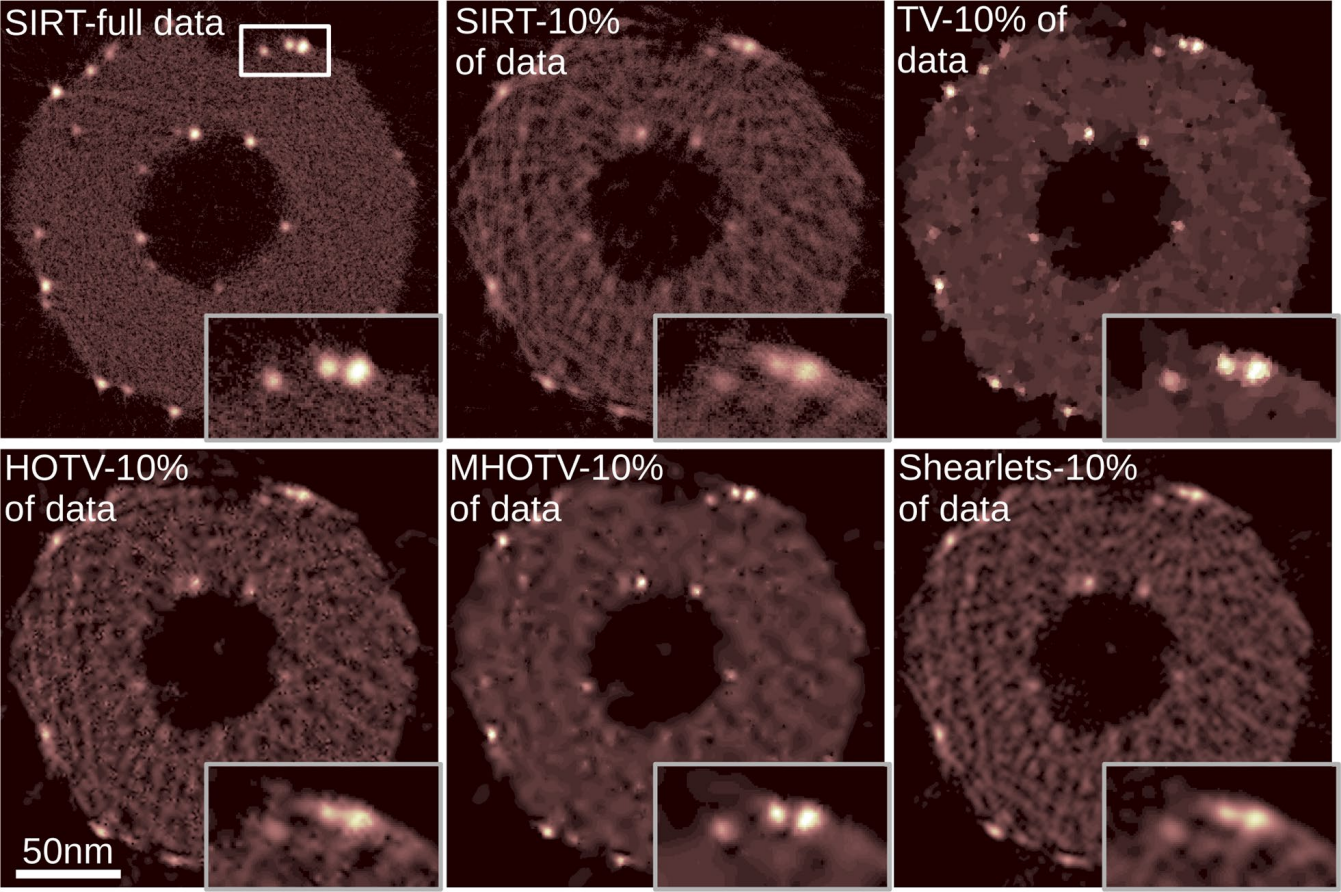
The 2D tomographic reconstruction of a single cross section of the 3D object visualized by a 2D projection in Fig. 4. A small patch is magnified in the bottom right of each image, where this patch is indicated in the top left image. A total of 180 projections are available for the reconstruction (resulting in the reconstruction shown in the top left), and the remaining reconstructions shown are from limiting the data to only 18 projections, i.e., only 10% of the original data

**Fig. 6:**
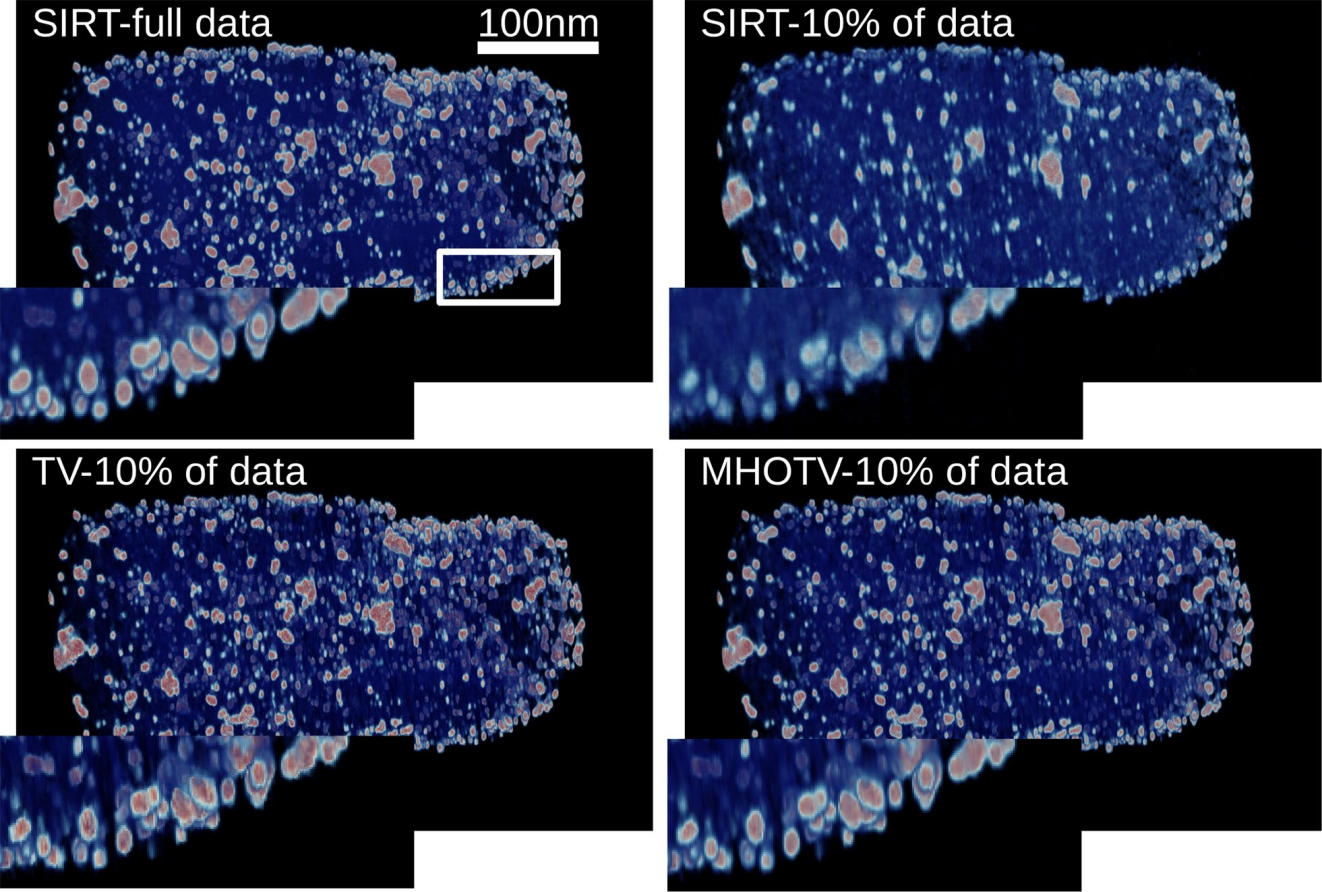
Shown is the volume rendering of the 3D tomographic reconstruction of the projected objected shown in Fig. 4. A small patch is magnified in the bottom left of each image, where this patch is indicated in the top left image. A total of 180 projections are available for the reconstruction (resulting in the reconstruction shown in the top left), and the remaining reconstructions shown are from limiting the data to only 18 projections, i.e., only 10% of the original data

For the next experiment with this data set, we reconstruct the 3D volume of the object from the available tilt series. To see how this problem is formulated as a regularized reconstruction in the form of (1), see for instance [8]. The tilt series is “full”, in the sense that the range of angles is over $180^{\circ }$ taken at every $1^{\circ }$. A single 2D cross section from the 3D reconstruction is shown in Fig. 5, and the 3D volume visualizations are shown in Fig. 6 using the tomviz software [43]. The resulting reconstruction from the full tilt series using the iterative least squares method known as SIRT (with a nonnegativity constraint) is shown in the top left of the Fig. 5, and this can be considered somewhat as an ideal solution. A small patch is magnified in the bottom right of the image for closer inspection. For a more challenging problem, we reduce the available tilt series to 10% of the data from 180 projections taken at every $1^{\circ }$ to only 18 projection taken at every $10^{\circ }$. The results of tomographic reconstruction from only these data are given in the remaining images in Fig. 5 along with the magnified image region. It is clearly observed that a notable degradation in the image quality takes place with the SIRT reconstruction in this case. We observe that the order 3 HOTV reconstruction again contains similar oscillatory artifacts observed previously. The shearlets are used in an iterative scheme here similar to the other $\ell _1$ regularization, as opposed to a simple post processing procedure with hard thresholding used in the denoising case. In this case, the shearlets do not perform as well. The TV and MHOTV on the other hand yield results similar to that with the full data set. With closer inspection in the magnified patch, we see that the TV does somewhat over smooth these small particles again, where MHOTV does an excellent job in separating the structure, perhaps even more accurately than the full data solution. Inspecting the 3D volumes in Fig. 6 we observe similar effects. In particular, there is an obvious degradation of the solution from SIRT when limiting the data, and the TV and MHOTV solutions appear to mitigate this effect. On the other hand, the TV solution appears “blocky”, suffering from the well-known staircasing effect [14, 15].

**Fig. 7:**
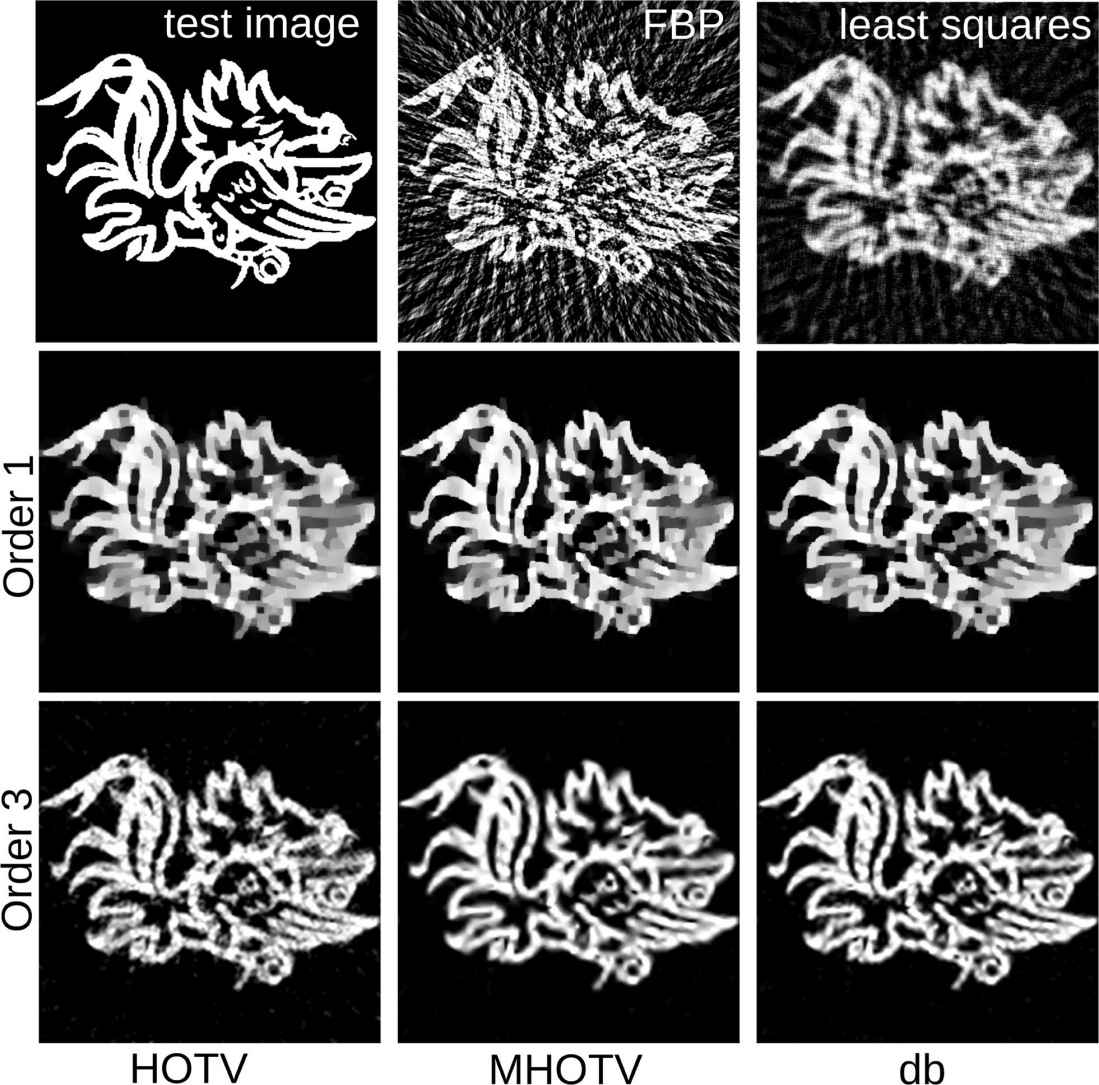
Reconstructions of phantom image from 29 tomographic projections. Orders 1 and 3 are shown for the regularization approaches. In the top right are the least squares and filtered backprojection (FBP) reconstructions for a baseline comparison

A simulated 2D tomographic image reconstruction was performed on the test image shown in Fig. 7, where the tomographic data are acquired with parallel beam geometry as in electron tomography. We simulated a total of 29 projection angles that are equally distributed across the full 180$^{\circ }$ angular range. Such a limited set of data are sometimes referred to as *limited data* tomography. Mean zero normally distributed noise was added to the data values, where the variance $\sigma ^2$ was set so that the SNR is 25.

Baseline comparisons are obtained by a conjugate gradient least squares solver and a filtered backprojection (FBP) reconstruction, which are shown in the figure. To ensure accurate comparison between the methods, the parameter $\lambda$ was set resulting in relative data errors, defined by $\Vert Af - b \Vert _2 / \Vert b\Vert _2$, to be all contained within an interval of size .0129.

As has been observed previously [8], due to a number of reasons including undersampling, noise, fine details between the image features, and nature of the regularization, the order 1 solutions (TV) can leave the fine features under resolved, even though the underlying image is truly a piecewise constant that classical TV was originally designed to recover. Each of these order 1 images appear relatively similar, with the MHOTV and Daubechies approaches showing modest improvements in resolving some features. As in the 1D case, the HOTV3 solution exhibits some small local oscillations that appear as noise in the image. However, this image, as well as the other order 3 approaches resolve the features notably more clear than the order 1 approaches. Both of the order 3 multiscale approaches appear less noisy than the HOTV order 3, while still maintaining a good approximation of the image features.

## Quantitative results

We performed two sets of simulations to compare the methods in a more quantitative manner. The first set of results presented here involved setting up 100 different test problems and then running all of our methods over each time for multiple noise levels, and the mean reconstruction error over all simulations is presented in Table 1, with the MHOTV resulting in the left of each column and Daubechies wavelets in the right of each column. It is important to note here that the parameter $\lambda$ in Eq. 8 was optimized in every reconstruction to yield the solution that minimized the true error between the test signal and the reconstruction, making for objective comparisons. To set up each test problem, a 1D piecewise quadratic polynomial (presumably ideal for order 3) was randomly generated over a 1024 stencil, and the entries in sampling matrix $A \in \mathbb {R}^{1024\times 1024}$ and added noise to *b* were randomly generated from a mean zero Gaussian distribution. Overall, these results show that MHOTV moderately outperforms Daubechies wavelets in each case, and remaining comparisons between the order and number of levels are generally consistent between MHOTV and the wavelets.

**Table 1:** Average relative reconstruction error over 100 simulations, as a function of the order of the method and number of levels in the multiscale approaches

SNR	Order 1		Order 2		Order 3		Order 1.5	Order 2.5
	mhotv	Daub	mhotv	Daub	mhotv	Daub	mhotv	mhotv
*2*								
1 level	.1624	.1624	.2039	.1961	.2464	.2306	.1819	.2328
2 levels	.1612	.1617	.1742	.1852	.2135	.2223	.1782	.2183
3 levels	.1699	.1615	*.1513*	.1778	.1745	.2149	.1776	.1975
4 levels	.2001	.1647	.1584	.1745	.1764	.2104	.2031	.2102
*5*								
1 level	.0864	.0864	.0971	.0914	.1293	.1090	.1025	.1287
2 levels	.0858	.0857	.0761	.0838	.0946	.1004	.0987	.1172
3 levels	.0926	.0864	*.0668*	.0805	.0766	.0982	.1016	.1133
4 levels	.1100	.0894	.0742	.0801	.0828	.0981	.1186	.1276
*10*								
1 level	.0543	.0542	.0509	.0480	.0694	.0572	.0690	.0841
2 levels	.0542	.0539	.0400	.0442	.0489	.0528	.0657	.0763
3 levels	.0589	.0547	*.0359*	.0430	.0399	.0522	.0694	.0776
4 levels	.0696	.0570	.0413	.0436	.0442	.0535	.0802	.0880

The minimums for each SNR are emphasized in italics

For the single-level case (original TV and HOTV), the error generally increases for higher orders, contrary to the results in previous work [20]. Multiple scales show notable improvement for the higher orders, whereas they show a mild reduction in accuracy for order 1. The most benefit for both orders 2 and 3 is seen when using 3 levels, and order 2 actually outperforms order 3. Finally, using the fact that (12) gives us a way to compute fractional orders of the method, we present also the results from orders 1.5 and 2.5. These are notably worse than the integer orders, a testament to the fact that these fractional-order derivates result in highly nonlocal differences.^4^

In the second set of results presented here, we ran a series of numerical simulations and measured the rate of successful recovery for each method as a function of the sampling rate. For each simulation, we randomly generated a piecewise polynomial of specified maximal degree over a 1024 stencil. This function was randomly sampled at the specified sampling rate precisely as in the previous 1D simulations in “Repeat of 1D simulations” section, where the sampling rate is defined by the number of samples divided by the number of grid points. Each regularization procedure was then used for reconstruction, and the $\ell _2$ error between the true function and reconstructed functions is determined. If the error was less than $10^{-2}$, then the reconstruction was said to yield a *successful recovery.* This simulation was carried out for each sampling rate in 20 trials, and the fraction of those 20 trials that yielded successful recovery is set as our probability of success. In each case, the generated test functions had five discontinuities, and the location of the jumps were drawn randomly from a uniform distribution on the approximation interval.

No noise was added for these simulations, as this can make the likelihood of an exact recovery unlikely. Therefore, for this case, our general $\ell _1$ model as a modification of (1) is given by

19 $$\begin{aligned} f_{rec} = \arg \min _f \Vert T f \Vert _1 \quad \text {s.t.} \quad Af = b, \end{aligned}$$

and similarly for our specific MHOTV model in (8). This constrained data fitting problem is solved by reformulating as an unconstrained problem with an augmented Lagrangian function [37, 44].

**Fig. 8:**
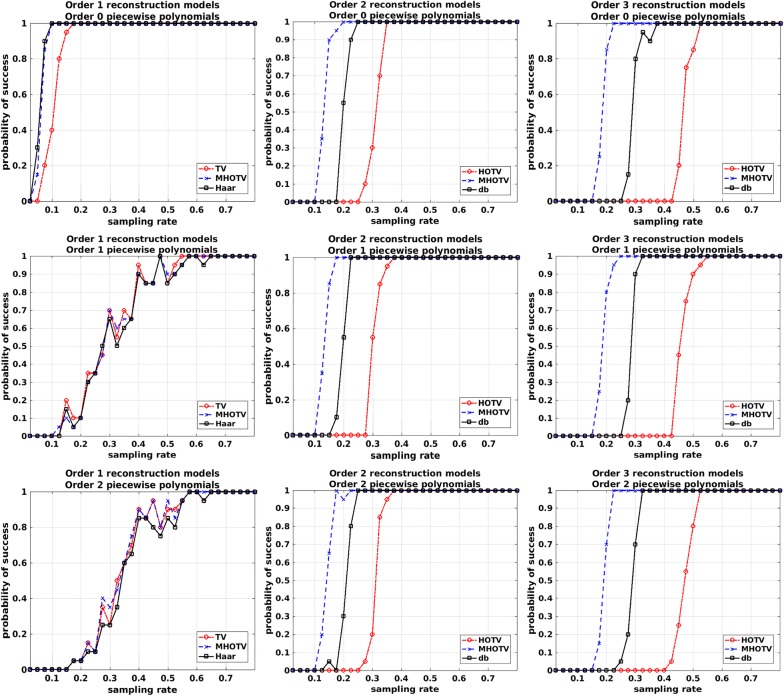
Probability of success for HOTV, MHOTV, and Daubechies wavelets at orders 1 (left column), 2 (middle column) and 3 (right column). A successful recovery is deemed whenever the relative $\ell _2$ error between the reconstruction and the true signal is less than $10^{-2}$. Top row: piecewise constant functions. Middle row: piecewise linear functions. Bottom row: piecewise quadratic functions

The results for these simulations are shown in Fig. 8. The results are separated in two ways, by the degree of the piecewise polynomial function that is sampled (varying along the rows) and the order of the regularization method (varying along the columns). In the first row are results for piecewise constant functions, in the second row are piecewise linear functions, and in the third row are piecewise quadratic functions. In all cases, the MHOTV yields the highest probability of success, regardless of the degree of the polynomial or order of the regularization, and the Daubechies wavelets success appears to generally lie somewhere between MHOTV and HOTV. The order 1 regularizations perform well only in the case of piecewise constant functions. On the other hand, the order 2 and 3 regularizations perform well for all function types, with order 2 again outperforming order 3 both with piecewise linear and quadratic signals.

## Conclusions

HOTV circumvents the staircasing often observed in TV solutions and has been shown to be more effective for problems with fine features, where resolution can be improved by increasing the order of derivatives in the regularization term [8]. In some applications, however, high-order derivatives promote solutions with spurious local oscillations, as shown in Fig. 1. The MHOTV regularization we introduce in this work is shown to mitigate unwanted oscillations while maintaining the resolution power of high-order regularization.

Although the theory for MHOTV reconstructions remains underdeveloped when compared to wavelets regularization [18, 30, 31, 45–48], our experiments indicate that MHOTV can outperform wavelets regularization in practical applications. Figure 3, for instance, shows fewer spurious oscillations in the MHOTV reconstruction than for Daubechies wavelets penalization, a feature that can also be observed for the 2D tomographic data and the experimental electron microscopy data. Moreover, our results show that MHOTV regularization requires fewer samples for successful reconstructions than for HOTV and wavelets. Computational efficiency is achieved by performing the transformation in Fourier space or by matrix decomposition, as derived in “Fast calculation of MHOTV operators” section. The associated matlab algorithms can be downloaded at [40], and some of the simulations in the proceeding section can also be found there.

## Appendix A: Proof of Theorem 1

### **Lemma 1**

*Let* $k,\ell \in \mathbb {Z}$ *with* $0\le \ell \le k$ *. Then we have the following Vandermonde-like identity:*

20 $$\begin{aligned} (-1)^p {k\atopwithdelims ()p} = \sum _{j=0}^\ell (-1)^j {k\atopwithdelims ()j} {k+1 \atopwithdelims ()\ell - j}, \end{aligned}$$

*where* $p = \ell /2$ *for* $\ell$ *even and* $p = (\ell - 1)/2$ *for* $\ell$ *odd.*

### Proof of Lemma 1

Consider the polynomial $p(x) = (1-x^2)^k (1+x)$, which can be factored as $p(x) = (1-x)^k (1+x)^{k+1}$. Both representations can be expanded using the binomial sum giving

21 $$\begin{aligned} p(x) = \sum _{j=0}^k (-x^2)^j {k \atopwithdelims ()j} (1+x) = \sum _{j=0}^k (-1)^j {k\atopwithdelims ()j}\left[ x^{2j} + x^{2j+1} \right] \end{aligned}$$

by the first representation and

22 $$\begin{aligned} p(x) = \left[ \sum _{j=0}^k (-x)^j {k\atopwithdelims ()j} \right] \left[ \sum _{j=0}^k x^j {k+1 \atopwithdelims ()j} \right] \end{aligned}$$

by the second representation. Since (21) and (22) are equivalent for all *x*, the coefficients of any particular power of *x* are equivalent, which is the equality we set out to prove. $\square$

### Proof of Theorem 1

Statement 3 is an immediate consequence of statement 2, since each matrix involved in the product is a convolution operator, and convolution operations are commutative and associative.

To prove statement 1, first observe that with increasing *m*, the nonzero entries in the rows of $P_m$ become increasingly spaced, and it easy to see that the general resulting product $P_m^{k+1}$ is essentially the same for each *m* with different spacings between the nonzero entries. Thus it is enough to show statement 1 for $m=1$. In the case $k=1$, this calculation can be checked directly. So suppose 1 holds for some arbitrary *k*. Then we need to show that $(P_1 P_1^{k+1})$ yields the desired result as defined by (16). It is fairly easy to see that the resulting entries of this product is simply the addition of two neighboring entries (modulo *N*) in $P_1^{k+1}$. Any such entries added together trivially yields the desired values, and the proper location of these values is also easy to confirm.

Similar arguments used for statement 1 also apply to statement 2. First, we can consider an inductive approach, over *m*, where we will need to show $\Phi _{k,2^{m+1}} = P_{m+1}^{k+1} \Phi _{k,2^{m}}$. Note that again due to the spacing of the entries of $P_m^{k+1}$, the argument for any arbitrary *m* is parallel to that for $m=1$, with just different handling of the indices. Therefore the case for $m=1$ suffices for the inductive step, and the case for $m=1$ is an immediate consequence of the previous lemma. $\square$

## Appendix B: Definitions

If $f,g\in \mathbb {R}^N$, then the convolution of *f* and *g* is given by

23 $$\begin{aligned} (f*g)_m = \sum _{n=0}^{N-1} f_n\, g_{m-n}, \quad \text {for}\, m = 0,1,\dots ,N-1, \end{aligned}$$

where for indices of *g* running outside of domain of *g*, a periodic extension of *g* is assumed. The discrete Fourier transform (DFT) of *f* is defined by

24 $$\begin{aligned} \mathcal {F}(f)_\xi = \sum _{n=0}^{N-1} f_n e^{\frac{-i2\pi }{N}n\xi } \quad \text {for}\, \xi = 0,1,\dots ,N-1, \end{aligned}$$

and the inverse discrete Fourier transform (IDFT) of *f* is given by

25 $$\begin{aligned} \mathcal {F}^{-1} (f)_n = \frac{1}{N} \sum _{\xi =0}^{N-1} f_\xi e^{\frac{i2\pi }{N}\xi n} \quad \text {for}\, n = 0,1,\dots ,N-1. \end{aligned}$$

## Abbreviations

ADMM: alternating direction method of multipliers
TV: total variation
HOTV: higher-order TV
MHOTV: multiscale HOTV
db: Daubechies [wavelets]
FFT: fast Fourier transform
DFT: discrete Fourier transform
CT: computed tomography
FBP: filtered backprojection
SIRT: simultaneous iterative reconstruction technique
SAR: synthetic aperture radar
CS: compressed sensing

## Footnotes

1 The number of samples is half the number of grid points.

2 This is akin to the dyadic scaling of the wavelet basis elements after the dyadic stretching.

3 For $\ell =n$ it is understood that the second sum is removed.

4 To observe these nonlocal stencils, one can compute the inverse Fourier transform of (12) for fractional orders *k*.

## References

[1] Rudin LI, Osher S, Fatemi E (1992). Nonlinear total variation based noise removal algorithms. Physica D Nonlinear Phenomena.

[2] Wei S-J, Zhang X-L, Shi J, Xiang G (2010). Sparse reconstruction for SAR imaging based on compressed sensing. Prog Electromagn Res.

[3] Bhattacharya, S., Blumensath, T., Mulgrew, B., Davies, M.: Fast encoding of synthetic aperture radar raw data using compressed sensing. In: IEEE 2007 IEEE/SP 14th workshop on statistical signal processing, pp. 448–452 (2007)

[4] Lustig M, Donoho D, Pauly JM (2007). Sparse MRI: the application of compressed sensing for rapid MR imaging. Magn. Resonance Med..

[5] Lysaker M, Lundervold A, Tai X-C (2003). Noise removal using fourth-order partial differential equation with applications to medical magnetic resonance images in space and time. IEEE Trans. Image Process..

[6] Ma, S., Yin, W., Zhang, Y., Chakraborty, A.: An efficient algorithm for compressed MR imaging using total variation and wavelets. In: IEEE conference on computer vision and pattern recognition, 2008. CVPR 2008, pp. 1–8 (2008). 10.1109/CVPR.2008.4587391

[7] Leary R, Saghi Z, Midgley PA, Holland DJ (2013). Compressed sensing electron tomography. Ultramicroscopy.

[8] Sanders T, Gelb A, Platte R, Arslan I, Landskron K (2017). Recovering fine details from under-resolved electron tomography data using higher order total variation regularization. Ultramicroscopy.

[9] Sanders T, Dwyer C (2017). Subsampling and inpainting approaches for electron tomography. Ultramicroscopy.

[10] King, E.J., Kutyniok, G., Lim, W.-Q.: Image inpainting: theoretical analysis and comparison of algorithms. In: SPIE optical engineering + applications, pp. 885–802 (2013)

[11] Eldar YC, Kutyniok G (2012). Compressed sensing: theory and applications.

[12] Candès E, Romberg J (2007). Sparsity and incoherence in compressive sampling. Inverse Probl..

[13] Candès EJ, Romberg J, Tao T (2006). Robust uncertainty principles: exact signal reconstruction from highly incomplete frequency information. IEEE Trans. Inf. Theory.

[14] Chan T, Marquina A, Mulet P (2000). High-order total variation-based image restoration. SIAM J. Sci. Comput..

[15] Blomgren P, Chan TF, Mulet P, Wong C-K (1997). Total variation image restoration: numerical methods and extensions. ICIP.

[16] Bredies K, Kunisch K, Pock T (2010). Total generalized variation. SIAM J. Imaging Sci..

[17] Hu Y, Jacob M (2012). Higher degree total variation (HDTV) regularization for image recovery. IEEE Trans. Image Process..

[18] Starck J-L, Murtagh F, Fadili JM (2010). Sparse image and signal processing: wavelets, curvelets, morphological diversity.

[19] Mallat S (2008). A wavelet tour of signal processing: the sparse way.

[20] Archibald R, Gelb A, Platte RB (2015). Image reconstruction from undersampled Fourier data using the polynomial annihilation transform. J. Sci. Comput..

[21] Stefan W, Renaut RA, Gelb A (2010). Improved total variation-type regularization using higher order edge detectors. SIAM J. Imaging Sci..

[22] Setzer S, Steidl G, Teuber T (2011). Infimal convolution regularizations with discrete l1-type functionals. Commun. Math. Sci..

[23] Chambolle A, Lions P-L (1997). Image recovery via total variation minimization and related problems. Numer. Math..

[24] Unser M, Fageot J, Ward JP (2017). Splines are universal solutions of linear inverse problems with generalized TV regularization. SIAM Rev..

[25] Steidl G, Didas S, Neumann J (2006). Splines in higher order TV regularization. Int. J. Comput. Vision.

[26] Scherzer O, Weickert J (2000). Relations between regularization and diffusion filtering. J. Math. Imaging Vision.

[27] Steidl G, Weickert J (2002). Relations between soft wavelet shrinkage and total variation denoising. Joint pattern recognition symposium.

[28] Steidl G, Weickert J, Brox T, Mrázek P, Welk M (2004). On the equivalence of soft wavelet shrinkage, total variation diffusion, total variation regularization, and sides. SIAM J. Numer. Anal..

[29] Kamilov U, Bostan E, Unser M (2012). Wavelet shrinkage with consistent cycle spinning generalizes total variation denoising. IEEE Signal Process. Lett..

[30] Guo K, Labate D (2007). Optimally sparse multidimensional representation using shearlets. SIAM J. Math. Anal..

[31] Kutyniok G (2012). Shearlets: multiscale analysis for multivariate data.

[32] Saghi Z, Benning M, Leary R, Macias-Montero M, Borras A, Midgley PA (2015). Reduced-dose and high-speed acquisition strategies for multi-dimensional electron microscopy. Adv. Struct. Chem. Imaging.

[33] Sanders T (2018). Parameter selection for HOTV regularization. Appl. Numer. Math..

[34] Daubechies I (1992). Ten lectures on wavelets.

[35] Coifman RR, Donoho DL (1995). Translation-invariant de-noising. Wavelets and statistics.

[36] Temizel A, Vlachos T, Visioprime W (2005). Wavelet domain image resolution enhancement using cycle-spinning. Electron. Lett..

[37] Li C, Yin W, Jiang H, Zhang Y (2013). An efficient augmented lagrangian method with applications to total variation minimization. Comput. Optim. Appl..

[38] Goldstein T, Osher S (2009). The split Bregman method for l1-regularized problems. SIAM J. Imaging Sci..

[39] Wang Y, Yang J, Yin W, Zhang Y (2008). A new alternating minimization algorithm for total variation image reconstruction. SIAM J. Imaging Sci..

[40] Sanders, T.: MATLAB Imaging algorithms: image reconstruction, restoration, and alignment, with a focus in tomography. http://www.toby-sanders.com/software, 10.13140/RG.2.2.33492.60801. Accessed 19 Aug 2016.

[41] Levin BD, Padgett E, Chen C-C, Scott M, Xu R, Theis W, Jiang Y, Yang Y, Ophus C, Zhang H (2016). Nanomaterial datasets to advance tomography in scanning transmission electron microscopy. Sci Data.

[42] Venkatakrishnan SV, Drummy LF, Jackson MA, De Graef M, Simmons J, Bouman CA (2013). A model based iterative reconstruction algorithm for high angle annular dark field-scanning transmission electron microscope (HAADF-STEM) tomography. IEEE Trans. Image Process..

[43] Levin BD, Jiang Y, Padgett E, Waldon S, Quammen C, Harris C, Ayachit U, Hanwell M, Ercius P, Muller DA (2018). Tutorial on the visualization of volumetric data using tomviz. Microscopy Today.

[44] Hestenes MR (1969). Multiplier and gradient methods. J. Optim. Theory Appl..

[45] Eck, M., DeRose, T., Duchamp, T., Hoppe, H., Lounsbery, M., Stuetzle, W.: Multiresolution analysis of arbitrary meshes. In: Proceedings of the 22nd annual conference on computer graphics and interactive techniques, ACM, New York, pp. 173–182 (1995).

[46] Tenoudji FC (2016). Wavelets; multiresolution analysis. Analog and digital signal analysis.

[47] Gao H-Y (1998). Wavelet shrinkage denoising using the non-negative garrote. J. Comput. Graph. Stat..

[48] Taswell C (2000). The what, how, and why of wavelet shrinkage denoising. Comput. Sci. Eng..

